# Duck Plague Virus pUL48 Protein Activates the Immediate-Early Gene to Initiate the Transcription of the Virus Gene

**DOI:** 10.3389/fmicb.2021.795730

**Published:** 2021-12-23

**Authors:** Tong Zhou, Dengjian Fan, Mingshu Wang, Anchun Cheng, Ying Wu, Qiao Yang, Bin Tian, Renyong Jia, Xumin Ou, Sai Mao, Di Sun, Shaqiu Zhang, Dekang Zhu, Shun Chen, Mafeng Liu, Xin-Xin Zhao, Juan Huang, Qun Gao, Yanling Yu, Ling Zhang

**Affiliations:** ^1^Institute of Preventive Veterinary Medicine, Sichuan Agricultural University, Chengdu, China; ^2^Key Laboratory of Animal Disease and Human Health of Sichuan Province, Sichuan Agricultural University, Chengdu, China; ^3^Avian Disease Research Center, College of Veterinary Medicine, Sichuan Agricultural University, Chengdu, China

**Keywords:** duck plague virus, pUL48, immediate early genes, transcriptional activation function, transcriptional activation domain

## Abstract

Duck plague caused by the duck plague virus (DPV) is an infectious disease that seriously harms the waterfowl breeding industry. The VP16 protein of α herpesvirus can bind to specific *cis*-acting elements upstream of the promoter of the immediate-early (IE, α) gene to promote the transcription of the IE gene, so it is also called the *trans*-inducer of IE gene (α-TIF). However, no studies on DPV α-TIF have been reported. This study investigated the DPV pUL48, a homolog of HSV-1 VP16, transcriptional activation region, target sequence, and viral protein affecting its transcriptional activation using a dual-luciferase reporter gene detection system, and pUL48 was identified as the α-TIF of DPV. (1) The regulation of pUL48 on DPV different gene promoters showed that pUL48 could activate all the promoters of IE genes (ICP4, ICP22, and ICP27) but not the promoters of early and late genes. (2) The activity of pUL48 to ICP4 and ICP22 promoters with different upstream lengths showed that pUL48 activated ICP4 and ICP22 promoters by acting on TAATGA (T) TAT element upstream of ICP4 promoter and TAATTATAT element upstream of ICP22 promoter, respectively. (3) Transcriptional activation of IE gene by truncated proteins of different lengths at the N-terminal of pUL48 was detected. The results showed that the transcriptional activation domain of pUL48 was amino acids 1–60 at the N-terminal, and amino acids 1–20 was its core region. In addition, it was found that pUL14, pUL46, and pUL47 significantly promoted the transcriptional activation of pUL48. The effects of loss of pUL47 and its nuclear localization signal on the nuclear entry and transcriptional activation function of pUL48 were further examined. The results showed that pUL47 could promote the nuclear entry of pUL48 through its nuclear localization signal at positions 40–50 and 768–777 amino acids, thus, enhancing the transcriptional activation function of pUL48 and synergistic promotion of viral gene transcription.

## Introduction

Duck plague virus (DPV) belongs to Herpesvirales, Herpesviridae Herpesvirales, Alphaherpesvirinae, Mardivirus, with typical herpesvirus particle structure (Guiping et al., 2007; Dhama et al., 2017; Lefkowitz et al., 2018). The DPV genome sequence has been published, including CHv strain, 2,085 strain, and VAC strain (Wu et al., 2012; Yang et al., 2014), whose genome has a typical herpesvirus type D genome structure, consisting of covalently bound unique long region (UL) and unique short region (US). Inverted repeat sequence (IRS) exists between UL and US, and terminal inverted repeat sequence (TRS) exists at US 3′. The UL-IRS-US-TRS structure is formed from 5′–3′ (Li et al., 2009; Wu et al., 2012). The DPV genome has 78 open reading frames (ORFs), including 65 ORFs in the UL region, 11 ORFs in the US region, and 2 ORFs in the IRS and TRS regions (Wang et al., 2011; Wu et al., 2012).

The 58-kDa *UL48* gene product, pUL48, is a homolog of HSV-1 VP16 and one of the most abundant proteins in the tegument of DPV. VP16 is a transcriptional activator of the IE gene of α herpesvirus, also known as an α-gene *trans*-inducing factor (α-TIF) (Zhang et al., 2016). After the herpesvirus infects host cells, the viral genome is transcribed in a specific sequence of IE gene, early gene (E, β), and late gene (L, γ), and this cascade of transcription mode is initiated by VP16 (Lu and Misra, 2000; Liu et al., 2015). VP16 released after virus infection interacts with host cell factor 1 (HCF-1), and octamer-binding factor 1 (Oct-1), through its DNA-binding domain (DBD), form a transcription regulatory complex that stably binds to the promoter of IE gene. Then the transcriptional activation domain (TAD) can recruit various transcription factors and promote correct initiation localization of RNA polymerase II to activate the transcription of the IE gene (LaBoissière et al., 1997; Lai and Herr, 1997). However, there is no report on DPV α-TIF. In this paper, the transcriptional activation of DPV pUL48, its transcriptional activation region, its target sequence, and the viral proteins affecting its transcriptional activation was studied by using the dual-luciferase reporter gene detection system.

## Materials and Methods

### Virus Strains, Duck Embryos, and Cells

The DPV-CHv strain (GenBank: JQ647509) of the duck plague virus was isolated and preserved by the Research Center for Poultry Disease Control and Prevention, Sichuan Agricultural University. The 10-day-old duck embryos were purchased from a duck farm in Pixian County, Sichuan Province, and used to make DEF cells. Taking 12-well plates as an example, each test used 5.86 × 10^5^ cells. Mouse ANTI-FLAG monoclonal antibody and mouse anti-HA monoclonal antibody were purchased from MBL. Rabbit anti-pUL48 polyclonal antibody serum and rabbit anti-pUL47 polyclonal antibody serum were prepared and preserved by the research Center of Poultry Disease Prevention and Control, Sichuan Agricultural University. Rabbit anti-beta (β)-actin antibody was obtained from Proteintech. HRP-conjugated goat anti-mouse IgG and HRP-conjugated goat anti-rabbit IgG were purchased from Beyotime.

### Species and Plasmid

Strains DH5α, eukaryotic expression plasmid pCAGGS, pCAGGS-pUL48-HA, pCAGGS-pUL14-Flag, pCAGGS-pUL46-Flag, pCAGGS-pUL47-Flag, pCAGGS-pUL49-Flag, pCDNA3.1-pUL47-Δ40–50 aa, pCDNA3.1-pUL47-Δ768–777 aa, pCDNA3.1-pUL47-Δ40–50 and 768–777 aa, pGL4-Basic, and double luciferase reporter plasmid pGL4-ICP4-Pro (740 bp), pGL4-ICP22-Pro (1,200 bp), pGL4-ICP22-Pro (1,500 bp), pGL4-UL15-Pro, pGL4-UL44-Pro, pGL4-ICP22-Pro, and pRL-TK were preserved and provided by the Research Center of Avian Disease Control, Sichuan Agricultural University.

### Dual-Luciferase Reporter Assay

Specific primers were designed (Table 1) and used for specific amplification and recovery of the amplification products. The promoter of each gene was cloned in the upstream of firefly luciferase gene by the chemiluminescence reaction of luciferase and substrate, and the luciferase recombinant reporter plasmid of each gene promoter was constructed. At the same time, in order to reduce the influence of internal variation factors on the accuracy of the experiment, pRL-TK plasmid with Renilla luciferase gene as the control plasmid was cotransfected with the reporter gene plasmid. After 24 h, the cells were lysed with 1 × cell lysis solution for 30 min and then freeze thawed at −80°C. Then 75-μl samples were added to the 96-well all-black plate, and 75 μl of Dual-Glo^®^ luciferase detection reagent was added to each well to generate a firefly fluorescence signal. The firefly luciferase reporter gene was first measured to obtain the *A*-value. After the fluorescence intensity of fireflies was quantified, 75 μl of Stop & Glo^®^ detection reagent was added to each well to quench the above reaction. At the same time, the Renilla luciferase reaction was started, and the fluorescence signal was detected to obtain the B value. For the instruction for specific determination, we referred to the Dual-Luciferase^®^ Reporter (DLR*™*) Assay System (Promega), and the data were processed (*A*-value/*B*-value).

**TABLE 1:** Design of luciferase reporter plasmids for different gene promoters.

The purpose gene	Primer	5′–3′ sequences
ICP4-Pro (900 bp)	ICP4-Pro-1 F	CTGGCCTAACTGGCCGGTACC ATATGGTTTAATTATAT
	ICP4-Pro-1 R	AGCTTGGCCGCCGAGGCCAGATCT GAGTAGAGCTGGGCGTCTATTA
ICP4-Pro (1,226 bp)	ICP4-Pro-2 F	CTGGCCTAACTGGCCGGTACC GCACTGCAGGATGTTTTA
	ICP4-Pro-2 R	ICP4-Pro-1 R
ICP4-Pro (1,235 bp)	ICP4-Pro-3 F	TCTCTGGCCTAACTGGCCGGTACC TAATGATATGCACTGCAGGATG
	ICP4-Pro-3 R	ICP4-Pro-1 R
ICP22-Pro (300 bp)	ICP22-Pro-1 F	TCTCTGGCCTAACTGGCCGGTACC TTGCTCAATACGGGA
	ICP22-Pro-1 R	AGCTTGGCCGCCGAGGCCAGATCT GTCTATCGCAGTTCT
ICP22-Pro (400 bp)	ICP22-Pro-2 F	TCTCTGGCCTAACTGGCCGGTACC
	ICP22-Pro-2 R	ICP22-Pro-1 R
ICP22-Pro (900 bp)	ICP22-Pro-3 F	TCTCTGGCCTAACTGGCCGGTACC CTCTAATGCATATTC
	ICP22-Pro-3 R	ICP22-Pro-1 R
ICP22-Pro (1,114 bp)	ICP22-Pro-4 F	TCTCTGGCCTAACTGGCCGGTACC CAGCGGTGCAGCCGT
	ICP22-Pro-4 R	ICP22-Pro-1 R
ICP22-Pro (1,200 bp)	ICP22-Pro-4 F	TCTCTGGCCTAACTGGCCGGTACC CCCCATCCCGATAGT
	ICP22-Pro-5 R	ICP22-Pro-1 R
ICP22-Pro (1,500 bp)	ICP22-Pro-6 F	TCTCTGGCCTAACTGGCCGGTACC AATCCAAAATGCCAG
	ICP22-Pro-6 R	ICP22-Pro-1 R
ICP27-Pro (1,000 bp)	ICP27-Pro-1 F	CTGGCCTAACTGGCCGGTACC TCACCTAATGGGGCCA
	ICP27-Pro-1 R	AGCTTGGCCGCCGAGGCCAGATCT GACGTTGGGCAGGTTCGTTA
ICP27-Pro (1,500 bp)	ICP27-Pro-2 F	CTGGCCTAACTGGCCGGTACC CATGAGTATAAGAATGCGATCGC
	ICP27-Pro-2 R	ICP27-Pro-1 R
UL28-Pro (495 bp)	UL28-Pro F	TCTCTGGCCTAACTGGCCGGTACC TGGGGCAACGCGTACAAT
	UL28-Pro R	AGCTTGGCCGCCGAGGCCAGATCT TTTCCAGATAGTGTTTG
TK-Pro (555 bp)	TK-Pro F	TCTCTGGCCTAACTGGCCGGTACC GCTGGGCTACAAATA
	TK-Pro R	AGCTTGGCCGCCGAGGCCAGATCT TTGATCGGAAATGTA
UL48-Pro (349 bp)	UL48-Pro F	TCTCTGGCCTAACTGGCCGGTACC TCTGGCCAGACCCCTCGT
	UL48-Pro F	AGCTTGGCCGCCGAGGCCAGATCT ATCCACACCCACACCTAT

### Western Blot

The supernatant was discarded 24 h after transfection, and the protein samples obtained by RIPA lysate (Strong) (Beyotime) were separated by 8% SDS-PAGE and then transferred to a PVDF membrane. Skim milk powder (5%) was used to block for 4 h at room temperature, and mouse ANTI-FLAG (1:4,000), mouse anti-HA (1:10,000), or rabbit anti-pUL48 (1:800) were used as primary antibodies to incubate overnight (4°C). Then HRP-conjugated goat anti-rabbit IgG (1:3,000) or HRP-conjugated goat anti-mouse IgG (1:3,000) was used as secondary antibodies and incubated at room temperature for 1 h with Western blotting (WB) and enhanced chemiluminescence (ECL) detection of the protein bands.

### Indirect Immunofluorescence Assay

The pCAGGS-pUL48-HA, pCAGGS-pUL47-Flag, pCDNA3.1-pUL47-Δ40–50 aa, pCDNA3.1-pUL47-Δ768–777 aa, and pCDNA3.1-pUL47-Δ40–50 and 768–777 aa plasmids were transfected into the cells on the cell slide according to the corresponding experimental groups. The cells were fixed with 4% paraformaldehyde 24 h after transfection, permeated at 4°C for 30 min with 0.25% Triton X-100, and sealed overnight with 5% BSA. Rabbit anti-HA (1:2,000), mouse ANTI-FLAG (1:2,000), mouse anti-HA (1:2,000), and rabbit anti-pUL47 (1:800) were incubated at 4°C overnight, then Alexa Fluor 568-labeled goat anti-rabbit/mouse IgG (1:1,000) and Alexa Fluor 488-labeled goat anti-mouse, and rabbit IgG (1:1,000) were incubated at room temperature for 1 h, respectively. The antibodies used were diluted with 1% BSA. Finally, the nucleus was stained with DAPI (1:1,000) at room temperature for 15 min, sealed with glycerol, and observed under an inverted fluorescence microscope (Zhao et al., 2019).

### RNA Isolation and RT-PCR

According to the instructions of the RNA extraction kit, reverse transcription reagent, and SYBR^®^Premix Ex TaqTM II Kit, RNA extraction, reverse transcription, and fluorescence quantitative PCR were performed successively to detect the mRNA levels of IE gene ICP4, ICP22, and ICP27. Quantitative primer sequences are shown in Table 2. β-actin was used as an internal reference, and the relative transcription level of IE gene in each group was analyzed by the 2^–ΔΔ^Ct relative quantitative method.

**TABLE 2:** Fluorescence quantitative PCR-related primers.

The purpose gene	Primer	5′–3′ sequences
ICP4	qF1	AATCTATGCCCGTCCAAGCTC
	qR1	CCCGGACCCATTACTAGGCACA
ICP22	qF2	CGTAGCGTCACATCAAGCAG
	qR2	GCGTTTGGTCCCTATAACCTC
ICP27	qF3	ACCTACAATTCAGCAACGCATA
	qR3	TGTTCGGAAGCCTCCATTCT
UL15	qF4	GCAGAGTGGCAAACCATACA
	qR4	TTCATTACCCGCTATCTCACC
UL23	qF5	GACTCTTGCGAACGCTTAATCC
	qR5	CAAGGGTATCCTCCGACAACA
UL28	qF6	TTAAGCTAAGCCGTGCCGAT
	qR6	TGGAGCCAGTTGTTGAAGCA
UL30	qF7	CATCATTTTGGCAAAGAATTCGCCACCA
	qR7	TGGCCATGACATCTATGGGTTACGC
UL44	qF8	GAAGGACGGAATGGTGGAAG
	qR8	AGCGGGTAACGAGATCTAATATTGA
UL48	qF9	GCGGGAAATATCATCACGACT
	qR9	CATTCAATAAGACGACGCCAT
β-actin	qF10	CCGGGCATCGCTGACA
	qR10	GGATTCATCATACT CCTGCTTGCT
UL30 probe		FAM-CCCTGGGTACAAGCG-MGB

### Construction of pUL48 Truncated Eukaryotic Expression Plasmid

Upstream and downstream primers were designed to specifically amplify the short fragment of pUL48 (Table 3). Finally, the primers and PrimeSTAR Max (TaKaRa) were used for specific amplification, and the amplification products were recovered. The pCAGGS vector was cut into linear fragments according to the restriction sites (Bgl II and EcoR I) selected during the design of primers. The restriction products were recovered, and homologous recombination was carried out using MonClone*™* Single Assembly Cloning Mix (Monad). Positive colonies were identified by PCR using 2 × Taq Master Mix (Vazyme), followed by Bgl II and EcoR I double digestion. After successful identification, positive clones were sent to Sangon Biotech for sequencing.

**TABLE 3:** Design of pUL48-truncated eukaryotic expression plasmid primer.

The purpose gene	Primer	5′–3′ sequences
pUL48 (Δ1–20 aa)	N-Δ1 F	CATCATTTTGGCAAAGAATTC GCCACCATGGCAGATCTAAGTG
	N-Δ1 R	TTGGCAGAGGGAAAAAGATCTCTA AGCGTAGTCTGGGACGTCGTATGG GTAATTATCTGGCGAGAACA
pUL48 (Δ1–40 aa)	N-Δ2 F	CATCATTTTGGCAAAGAATTCGCCAC CATGGCCATGCGGACTTAAGTGCAG
	N-Δ2 R	N-Δ1 R
pUL48 (Δ1–60 aa)	N-Δ3 F	CATCATTTTGGCAAAGAATTCGCCACC ATGGCCATGGAACTAAGCTTAGAAGGG
	N-Δ3 R	N-Δ1 R
pUL48 (Δ1–80 aa)	N-Δ4 F	CATCATTTTGGCAAAGAATTCGCCACCA TGGCCATGACATCTATGGGTTACGC
	N-Δ4 R	N-Δ1 R
pUL48 (Δ1–100 aa)	N-Δ5 F	CATCATTTTGGCAAAGAATTCGCCACC ATGGCCATGTCACCGGCTCAGTTTTC
	N-Δ5 R	N-Δ1 R
pUL48 (Δ21–40 aa)	N-Δ6 F	CATCATTTTGGCAAAGAATTCGCCACC ATGGATACATTTGATGAACTCGACAGG CTACTGGCTAATATTGACGCGTATTCA GCAATGACGGACTTAAGTGCAGAA
	N-Δ6 R	N-Δ1 R
pUL48 (Δ41–60 aa)	N-Δ7 F1	CATCATTTTGGCAAAGAATTCGCCACC ATGGATACATTTGATGAACTCGACAGGCT
	N-Δ7 R1	TCCTTCTGCACTTAAGTCCGT CGCCTGTGCAGTATAACC
	N-Δ7 F2	GGTTATACTGCACAGGCG GAACTAAGCTTAGAAGGGAAG
	N-Δ7 R2	N-Δ1 R

## Results

### pUL48 Could Effectively Activate the Promoter of the Intermediate-Early Gene but Had No Effect on the Promoter Activity of the E Gene or L Gene (Including Itself)

**FIGURE 1:**
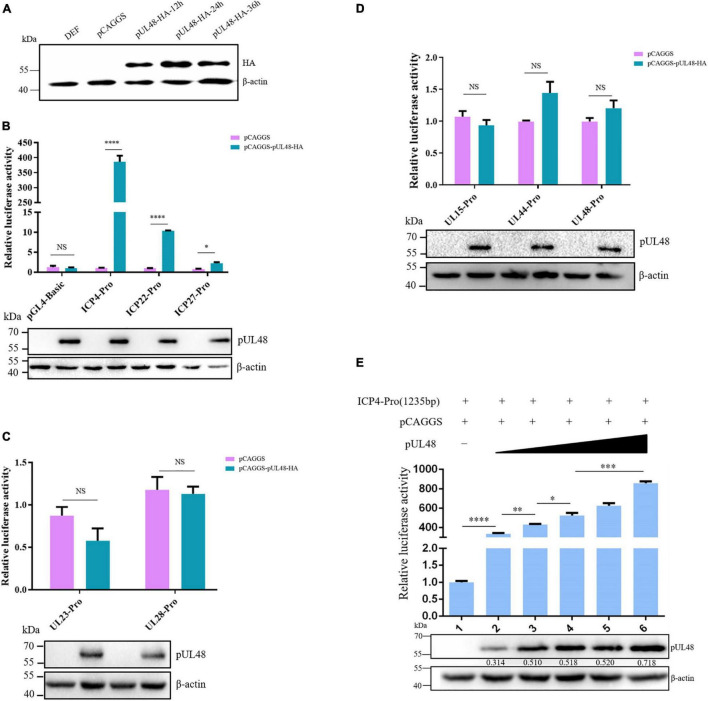
Regulation of pUL48 on duck plague virus (DPV) gene promoters of different types. **(A)** Expression of pCAGGS-pUL48-HA in DEF. pCAGGS and pCAGGS-pUL48-HA were transfected into DEF, respectively, and cell protein samples were collected at 12, 24, and 36 h for Western blot. **(B)** The regulation of pUL48 on the IE gene promoter of DPV. **(C)** The regulation of pUL48 on DPV early gene promoters. **(D)** The regulation of pUL48 on DPV late gene promoter. **(E)** Dose-dependent activation of pUL48 on ICP4-Pro (1,235 bp). pGL4-basic, ICP4-Pro-Luc, ICP22-Pro-Luc, ICP27-Pro-Luc, UL23-Pro-Luc, UL28-Pro-Luc, UL15-Pro-Luc, UL44-Pro-Luc, and UL48-Pro-Luc were cotransfected into DEF with pCAGGS, pCAGGS-pUL48-HA, and pRL-TK/0.05, 0.1, 0.2, 0.3, and 0.4 μg pCAGGS-pUL48-HA were cotransfected into DEF with pCAGGS, ICP4-Pro (1,235 bp)-Luc and pRL-TK. Except for the reference plasmid pRL-TK, the transfection ratio of other plasmids was 1:1:1, and the transfection quantity of pRL-TK was 1/20 of the double luciferase plasmids; pCAGGS was a blank group. Cell samples were collected 24 h after transfection. The activity of each promoter was detected by a dual-luciferase reporting system. The corresponding concentration of pCAGGS-pUL48-HA was transfected into DEF. At 24 h after transfection, cell protein samples were collected for Western blot analysis. Student’s *t*-test was used to analyze the differences of the two groups. The significance was as follows: NS showed no difference, *p < 0.05, ^**^p < 0.01, ^***^p < 0.001, ^****^p < 0.0001.

The Dual-Luciferase reporter system was used to detect transcriptional activation of DPV gene promoters of different types to explore whether DPV pUL48 has a transcriptional activation function. First, the recombinant reporter plasmids pGL4-ICP4-Pro (1,235 bp)-Luc, pGL4-ICP22-Pro (1,114 bp)-Luc, pGL4-ICP27-Pro (1,000 bp)-Luc, pGL4-UL23-Pro (555 bp)-Luc, pGL4-UL28-Pro (495 bp)-Luc, and pGL4-UL48-Pro (349 bp)-Luc were constructed by using the pGL4-Basic vector. Western blot confirmed the normal expression of eukaryotic plasmid pCAGGS-pUL48-HA in DEF cells, about 58 kDa (Figure 1A). The results showed that transposition of pUL48 with luciferin reporter plasmid of ICP4, ICP22, and ICP27 promoter of IE gene could promote the expression of luciferin protein downstream of the promoter fluorescence intensity of the reaction with the substrate was higher than that of the control group. The results showed that pUL48 could effectively activate the promoter of IE gene (Figure 1B), while the fluorescence intensity measured by pUL48 and the luciferase reporter plasmid of E or L gene promoter had no significant difference with the control group, indicating that pUL48 had no effect on the promoter activity of E or L gene (including itself) (Figures 1C,D). Therefore, pUL48 is the α-TIF of the DPV IE gene.

In addition, through exploring the dose-dependent relationship of pUL48 transcriptional activation, it was found that there was a certain dose-dependent relationship between the expression level of pUL48 and its activation effect on ICP4-Pro (1,235 bp), but only a little expression of pUL48 could significantly improve the activity of ICP4-Pro (1,235 bp) (Figure 1E). These results indicated that even low expression of pUL48 could regulate promoter activity.

### pUL48 Acts on *cis*-Acting Elements Upstream of Immediate-Early Gene Promoter

**FIGURE 2:**
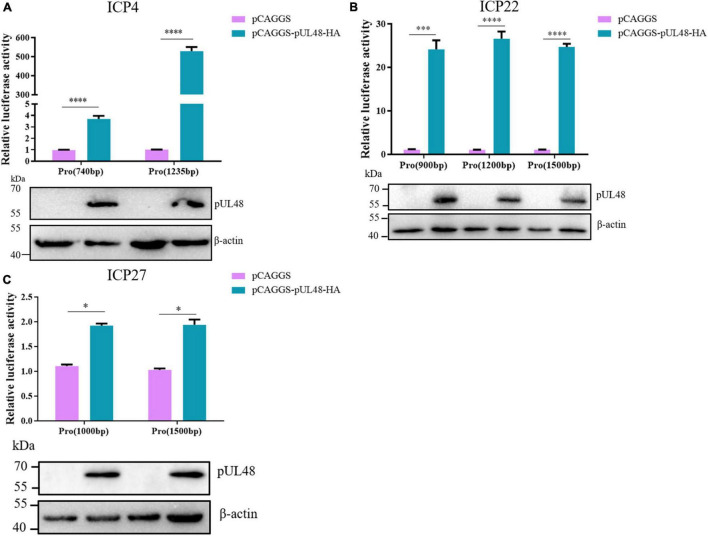
Regulation of pUL48 on promoters of different lengths of intermediate-early (IE) gene. The regulation of pUL48 on ICP4/ICP22/ICP27 promoters of different lengths. ICP4-Pro (740 bp)-Luc, ICP4-Pro (1,235 bp)-Luc, ICP22-Pro (900 bp)-Luc, ICP4-Pro (1,200 bp)-Luc, ICP4-Pro (1,500 bp)-Luc, ICP27-Pro (100 bp)-Luc, and ICP27-Pro (1,500 bp)-Luc were cotransfected into DEF with pCAGGS and pCAGGS-pUL48-HA and pRL-TK, respectively. The transfection ratio of pCAGGS and pCAGGS-pUL48-HA with each double luciferase plasmid was 1:1, and the transfection quantity of pRL-TK was 1/20 of each double luciferase plasmid. Cell samples were collected 24 h after transfection. The activity of each promoter was detected by the dual-luciferase reporting system. pCAGGS-pUL48-HA was transfected into DEF. At 24 h after transfection, cellular protein samples were collected for Western blot analysis. Student’s *t*-test was used to analyze the differences of the two groups. The significance was as follows: *p < 0.05, ^***^p < 0.001, ^****^p < 0.0001.

Figure 1 shows that pUL48 activated the three IE gene promoters in different degrees. To further determine whether the activation effect of pUL48 on IE gene promoter was related to promoter length, the activation effect of pUL48 on promoters of IE gene of different lengths was detected in this study. The results showed that pUL48 had significantly different activation effects on ICP4 promoter with different lengths, and the activation effect on ICP4-Pro (1,235 bp) was much higher than that on ICP4-Pro (740 bp) (Figure 2A). The activation of ICP22 promoter with different lengths (900/1,200/1,500 bp) showed obvious effects (Figure 2B). Activation of ICP27 promoters of different lengths (1,000/1,500 bp) was weak (Figure 2C).

**FIGURE 3:**
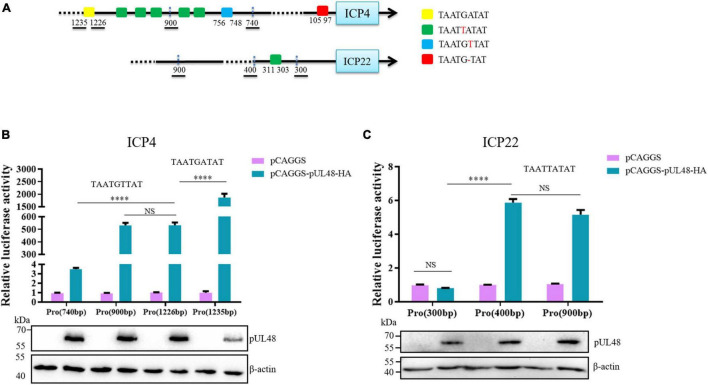
The regulatory effects of pUL48 on ICP4 and ICP22 promoters containing different components. **(A)** Initial upstream *cis*-element sequence analysis of ICP4 and ICP22. Different colored squares represent different possible sequences of *cis*-elements. **(B)** The regulation of pUL48 on ICP4 promoter containing different elements. **(C)** The regulation of pUL48 on ICP22 promoter containing different elements. ICP4-Pro (740 bp)-Luc, ICP4-Pro (900 bp)-Luc, ICP4-Pro (1,226 bp)-Luc, ICP4-Pro (1,235 bp)-Luc, ICP22-Pro (300 bp)-Luc, ICP22-Pro (400 bp)-Luc, ICP22-Pro (900 bp)-Luc, pCAGGS, pCAGGS-pUL48-HA, and pRL-TK were cotransfected into DEF. At 24 h after transfection, cell samples were collected, and the activity of each promoter was detected by the dual-luciferase reporting system. pCAGGS-pUL48-HA was transfected into DEF. At 24 h after transfection, cell protein samples were collected for Western blot analysis. Student’s *t*-test was used to analyze the differences of the two groups. NS was not significant, ^****^p < 0.0001.

The above results indicated that the *cis*-elements activated by pUL48 exits between 740 and 1,235 bp upstream of the ICP4 promoter. A possible *cis*-acting element was found by comparing the upstream sequences of DPV ICP4 and ICP22 promoter with the reported *cis*-acting element sequences of VP16 in other herpesviruses (Figure 3A). Depending on the location of the *cis*-elements, the luciferase reporter plasmids pGL4-ICP4-Pro (1,235 bp), pGL4-ICP4-Pro (740 bp), and pGL4-ICP22-Pro (900 bp) used in the above experiments and the constructed pGL4-ICP4-Pro (1,226 bp), pGL4-ICP4-Pro (900 bp), pGL4-ICP22-Pro (300 bp), pGL4-ICP22-Pro (400 bp), and pGL4-ICP27-Pro (1,500 bp) luciferase reporter plasmid were used to determine the *cis*-acting element of pUL48.

The detection results of pUL48 regulating ICP4 promoter with different components showed that the activity of ICP4-Pro (1,235 bp) was increased nearly 2,000 times under pUL48, which was much higher than that of pUL48 on ICP4-Pro (1,226 bp), indicating that TAATGATAT was a *cis*-element highly suitable for pUL48 transcriptional activation. The activity of ICP4-Pro (1,226 bp) and ICP4-Pro (900 bp) increased about 500 times under pUL48, and there was no difference between them. These results indicated that the TAATTATAT repeat unit existing between 756 and 1,226 bp upstream of ICP4 was not the sequence of pUL48 acting on the ICP4 promoter. The activity of ICP4-Pro (740 bp) was only three times higher than that of ICP4-Pro (900 bp) under pUL48, which was far lower than that of ICP4-Pro (900 bp), suggesting that the TAATGTTAT sequence at 748 and 756 bp upstream of ICP4 promoter was also the *cis*-element of pUL48 acting on ICP4 (Figure 3B). The results showed that pUL48 had no effect on the activity of ICP22-Pro (300 bp) but had the same activation effect on ICP22-Pro (400 bp) and ICP22-Pro (900 bp). The TAATTATAT sequence at 303–311 bp upstream of ICP22 promoter was the *cis*-element of pUL48 acting on ICP22 (Figure 3C).

### pUL48 N-Terminal 1–20 Amino Acids Are the Key Region of Its Transcriptional Activation Domain

**FIGURE 4:**
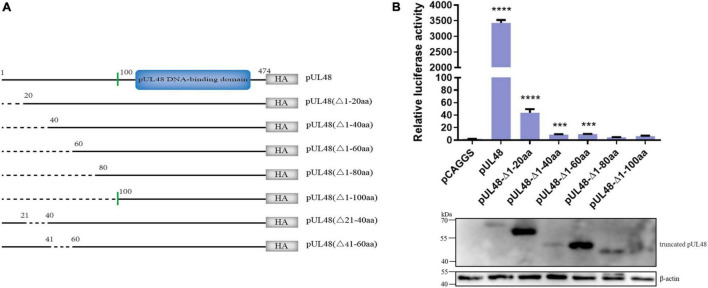
Effect of pUL48-truncated protein on ICP4 promoter activity. **(A)** Structure diagram of N-terminal-truncated protein of pUL48. **(B)** Regulatory effects of pUL48 N-terminal-truncated protein on ICP4-Pro (1,235 bp). The pUL48 N-terminal-truncated plasmid was cotransfected with ICP4-Pro (1,235 bp) and pRL-TK into DEF. At 24 h after transfection, cell samples were collected, and the activity of each promoter was detected by the dual-luciferase reporting system. The pUL48 N-terminal-truncated plasmid was transfected into DEF. At 24 h after transfection, cell protein samples were collected for Western blot analysis. Student’s *t*-test was used to analyze the control and the differences of each experimental group. ***p < 0.001, ****p < 0.0001.

Considering that pUL48 has a strong activation effect on ICP4-Pro (1,235 bp), the article further constructed the pUL48 truncated eukaryotic expression plasmids of pUL48 (Δ1–20 aa), pUL48 (Δ1–40 aa), pUL48 (Δ1–60 aa), pUL48 (Δ1–80 aa), pUL48 (Δ1–100 aa), pUL48 (Δ21–40 aa), and pUL48 (Δ41–60 aa), and preliminarily screened the transcriptional activation domain using ICP4-Pro (1,235 bp). The results show that the activation of pUL48 (Δ1–60 aa) to ICP4-Pro (1,235 bp) is significantly inhibited, while pUL48 (Δ1–80 aa) and pUL48 (Δ1–100 aa) completely lose the activation of ICP4-Pro (1,235 bp) (Figure 4). Therefore, the transcriptional activation domain of pUL48 was determined to be located at 1–60 aa of its N-terminal.

**FIGURE 5:**
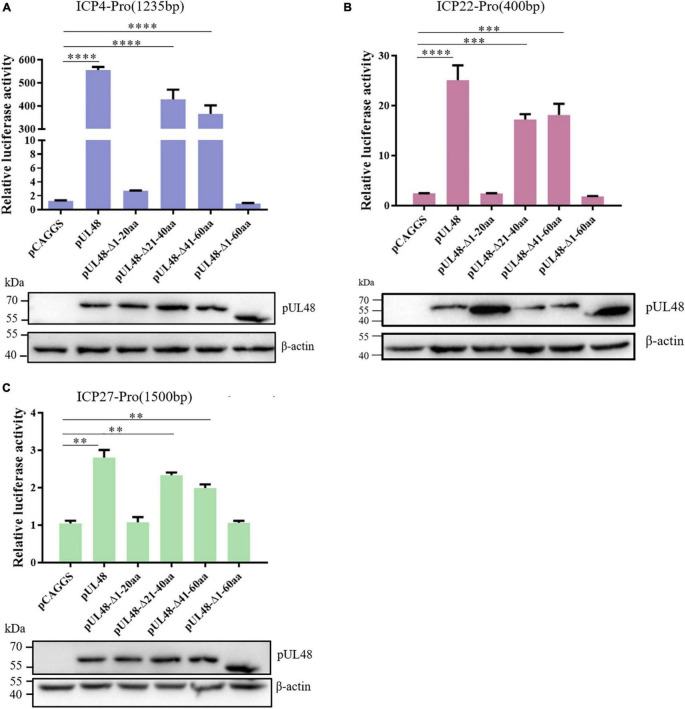
Identification of the core region of the DPV pUL48 transcriptional activation domain. The regulation of ICP4/ICP22/ICP27 promoter by a deletion in different regions of the pUL48 transcriptional activation domain. The N-terminal-truncated plasmids of pUL48 were cotransfected into DEF with ICP4-Pro (1,235 bp), ICP22-Pro (900 bp), ICP27-Pro (1,500 bp), and pRL-TK. pCAGGS was the negative control, and pCAGGS-pUL48-HA was the positive control. Cell samples were collected 24 h after transfection, and the activity of each promoter was detected by a dual-luciferase reporting system. The pUL48 N-terminal-truncated plasmid was transfected into DEF. At 24 h after transfection, cellular protein samples were collected for Western blot analysis. Student’s *t*-test was used to analyze the differences of the two groups, and the significance was as follows: ^**^p < 0.01, ^***^p < 0.001, ^****^p < 0.0001.

In order to further determine the core region of the pUL48 transcriptional activation domain, the regulation of pUL48-truncated proteins—pUL48 (Δ1–20 aa), pUL48 (Δ21–40 aa), and pUL48 (Δ41–60 aa) on the IE gene promoter was detected. The results showed that deletion of pUL48 N-terminal 1–20 aa or 1–60 aa had the same effect, which significantly inhibited the activation of IE gene promoter by pUL48, while deletion of N-terminal 21–40 aa or 41–60 aa only slightly reduced the activation of all IE gene promoters (Figure 5), indicating that 1–20 aa at the N-terminal of pUL48 is the key region of its transcriptional activation domain (1–60 aa at the N-terminal).

**FIGURE 6:**
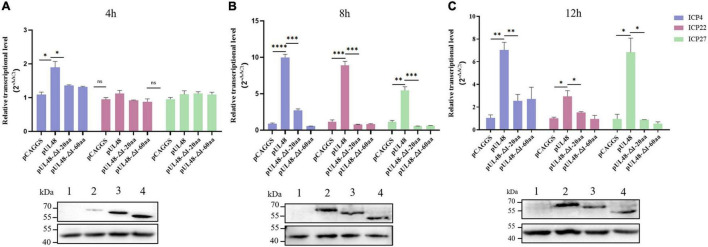
Effect of overexpression of pUL48 and its truncated protein on viral IE gene transcription. The relative transcription level of the IE gene at 4/8/12 h after infection with MOI = 1 DPV-CHv; pCAGGS, pCAGGS-pUL48-HA, pCAGGS-pUL48 (Δ1–20 aa)-HA, and pCAGGS-pUL48 (Δ1–60 aa)-HA were transfected into a 12-well plate DEF, respectively. At 12 h after transfection, DEF was infected with DPV CHv strain with MOI = 1. The virally infected cells were collected 4, 8, and 12 h after infection into the non-RNA enzyme EP tube using RNAiso Plus. According to the instructions of the RNA extraction kit, reverse transcription reagent and SYBR^®^Premix Ex Taq*™* II Kit, RNA extraction, reverse transcription, and quantitative fluorescence PCR were performed successively to detect the mRNA levels of IE gene ICP4, ICP22, and ICP27. The expression of each truncated protein of pUL48 was detected by WB. 1, pCAGGS; 2, pUL48; 3, pUL48 (Δ1–20 aa); 4: pUL48 (Δ1–60 aa). Relative quantitative test results were processed using 2^– ΔΔ^Ct, and the results were normalized to the control group. Student’s *t*-test was used to analyze the experimental and control groups differences. *p < 0.05, ^**^p < 0.01, ^***^p < 0.001, ^****^p < 0.0001.

The influence of overexpression of pUL48 or its truncated protein on the transcription level of the IE gene after virus infection was further detected. The results showed that overexpression of pUL48 significantly increased the transcription level of all IE genes, with the most effect at 8 h compared with 4 and 12 h (Figure 6), indicating that pUL48 played its transcriptional activation function at the early stage of the DPV genome transcription. Overexpression of pUL48 (Δ1–20 aa) only increased the transcription level of ICP4, and there was no difference between ICP22 and ICP27 compared with the control group. Overexpression of pUL48 (Δ1–60 aa) also did not promote the transcription of ICP4, again demonstrating that the activation domain of pUL48 was located at the N-terminal 1–60 aa, and 1–20 aa is its core region.

### pUL47 Enhanced the Transcriptional Activation of pUL48 by Promoting Its Entry Into the Nucleus

**FIGURE 7:**
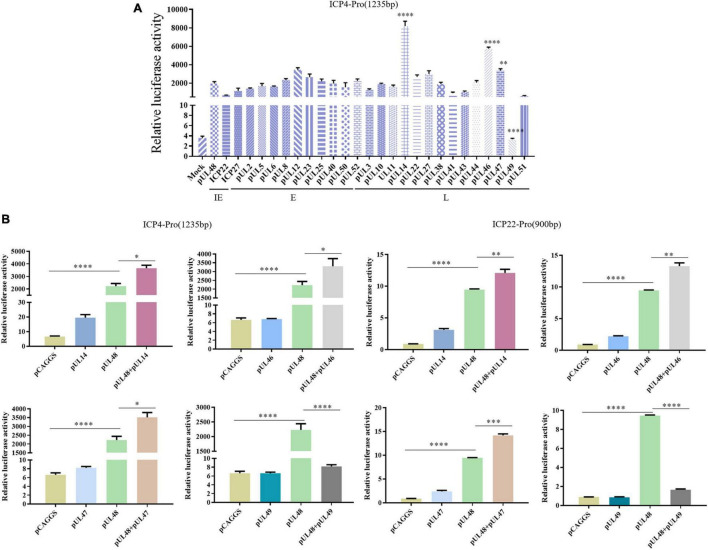
Screening of viral proteins affecting the transcriptional activation of pUL48. **(A)** Various viral proteins affected the activation of pUL48 on ICP4-Pro (1,235 bp). The Mock: pCAGGS + ICP4-Pro (1,235 bp); Control: pUL48 + ICP4-Pro (1,235 bp); ICP22: ICP22 + pUL48 + ICP4-Pro (1,235 bp). The other groups were set up in the same way as ICP22. Will pGL4-ICP4-Pro(1235 bp)-Luc, reference plasmids pRL-TK, and pCAGGS-pUL48-HA were cotransfected with 26 eukaryotic expression plasmids of DPV protein, including pCAGGS-UL2-Flag and pCAGGS-UL47-Flag, respectively (the control group was transfected with the same amount of pCAGGS without loading), and the samples were collected at 24 h. The relative fluorescence intensity of the transfected virus protein group and the control group was detected and compared with screen out, the viral target protein that affected the transcriptional activation function of pUL48. **(B)** The activation of ICP4 and ICP22 promoters by pUL48 was regulated by pUL14/pUL46/pUL47/pUL49. It was verified that the target protein did not affect the IE gene promoter but regulated the transcriptional activation function of pUL48. Student’s *t*-test was used to analyze the differences of the two groups. *p < 0.05, ^**^p < 0.01, ^***^p < 0.001, ^****^p < 0.0001.

After the transcriptional activation function and the transcriptional activation domain of pUL48 were determined, the viral proteins affecting the transcriptional activation function of pUL48 were further screened. The results showed that other viral proteins could regulate the transcriptional activation function of pUL48. Among them, pUL14, pUL46, and pUL47 could enhance the activation effect of pUL48 on the ICP4 promoter, while pUL49 could inhibit the activation effect (Figure 7A).

To determine whether the target protein regulates ICP4 promoter activity by enhancing or inhibiting the transcriptional activation function of pUL48 rather than acting on it, the effects of these viral proteins on promoter activity alone were examined. The results showed that pUL14, pUL46, and pUL47 had no significant effect on ICP4 and ICP22 promoters, mainly enhancing the transcriptional activation function of pUL48, while pUL49 had a significant inhibitory effect (Figure 7B).

**FIGURE 8:**
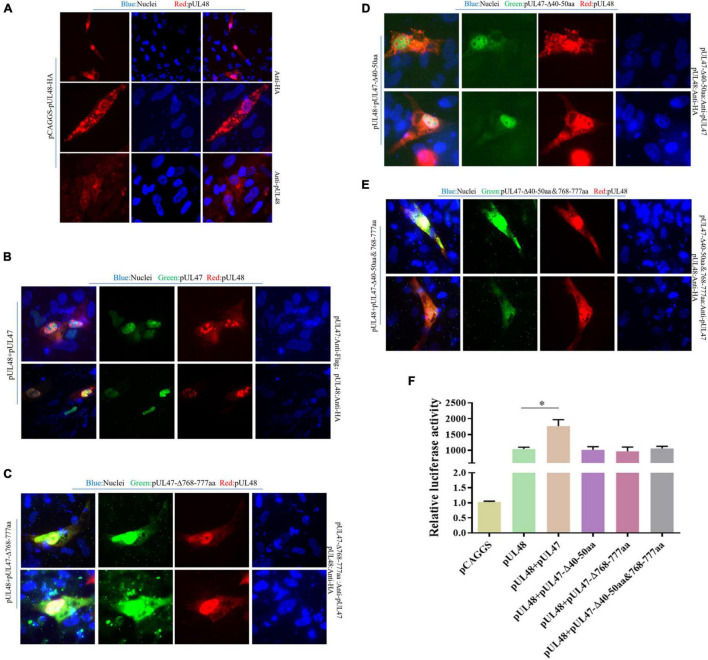
Effects of pUL47 NLS on transcriptional activation of pUL48. **(A)** Cell localization of pUL48. **(B)** The effect of pUL47 on the nucleation of pUL48. **(C)** The influence of pUL47-Δ40–50 aa on pUL48 localization. **(D)** The influence of pUL47-Δ768–777 aa on pUL48 localization. **(E)** The influence of pUL47-Δ40–50 and 768–777 aa on pUL48 localization. First, pCAGGS-pUL48-HA was transfected separately. Then pCAGGS-pUL48-HA, pCAGGS-pUL47-Flag, pcDNA3.1-pUL47-Δ40–50 aa, pcDNA3.1-pUL47-Δ768–777 aa, and pcDNA3.1-pUL47-Δ40–50 and 768–777 aa with NLS deletion plasmids were cotransfected according to corresponding experimental groups, and indirect immunofluorescence assay was performed. **(F)** Effect of pUL47 NLS deletion on ICP4-Pro (1,235 bp) activation of pUL48. pCAGGS, pCAGGS-pUL48-HA, pCAGGS-UL47-Flag, pcDNA3.1-pUL47-Δ40–50 aa, pcDNA3.1-pUL47-Δ768–777 aa, and pcDNA3.1-pUL47-Δ40–50 and 768–777 aa were cotransfected into DEF with pGL4-ICP4-Pro (1,235 bp)-Luc and pRL-TK, except the reference plasmid pRL-TK. The transfection ratio of other plasmids with pGL4-ICP4-Pro (1,235 bp)-Luc was 1:1, and the transfection quantity of pRL-TK was 1/20 of pGL4-ICP4-Pro (1,235 bp)-Luc. Cell samples were collected 24 h after transfection, and ICP4-Pro (1,235 bp) promoter activity was detected by a dual-luciferase reporting system. Student’s *t*-test was used to analyze the differences of the two groups; *p < 0.05.

Due to previous reports that pUL14, pUL46, and pUL47 all have nuclear localization signal (NLS) (Li et al., 2016; He et al., 2018; Xu et al., 2020), therefore, in this paper, DPV pUL47 and its recombinant plasmids deleted their NLSs (40–50 and 768–777 aa), which were used to explore whether the regulation of DPV pUL14, pUL46, and pUL47 on the transcriptional activation function of pUL48 was realized by affecting the entry of pUL48 into the nucleus. The results showed that pUL48 was dispersed in the cytoplasm and the nucleus when transfected alone and was mainly located in the cytoplasm (Figure 8A). However, after coexpression of pUL48 and pUL47, pUL48 presented a dense point-like distribution in the nucleus (Figure 8B). When pUL48 was coexpressed with pUL47 NLS single-deletion plasmid pUL47-Δ768–777 aa (Figure 8C) and pUL47-Δ40–50 aa (Figure 8D) or pUL47 NLS double-deletion plasmid pUL47-Δ40–50 and 768–777 aa (Figure 8E), pUL48 was no longer distributed in the nucleus but was dispersed in the nucleus and cytoplasm.

Subsequently, to determine whether pUL47 synergistically enhanced the transcriptional activation function of pUL48 by promoting the entry of pUL48 into the nucleus, the effect of pUL47 NLS deletion on pUL48 transcriptional activation function was further detected. The results showed that ICP4-Pro (1,235 bp) promoter activity was increased less than 1,000 times during pUL48 single expression, while ICP4-Pro (1,235 bp) promoter activity was increased nearly 1,500 times after pUL47 and pUL48 coexpression, indicating that ICP4-Pro (1,235 bp) was further activated by the addition of pUL47.There was no significant difference in the activity of ICP4-Pro (1,235 bp) when the recombinant plasmids of pUL47-Δ40–50 aa, pUL47-Δ768–777 aa, pUL47-Δ40–50 and 768–777 aa, and pUL47-Δ40–50 and 768–777 aa were coexpressed with pUL48 and separately expressed pUL48 (Figure 8F), indicating that the deletion of pUL47 NLS did not enhance the activation effect of pUL48 on ICP4-Pro (1,235 bp). These results indicated that pUL47 could synergistically enhance the transcriptional activation function of pUL48 by promoting the entry of pUL48 into the nucleus.

## Discussion

In most α herpesviruses, VP16 is conserved. VP16 not only participates in the structure of the virus tegument but also binds to the expression of the viral gene upstream of the IE (α) gene promoter and is the *trans*-inducing factor (α-TIF) of the virus IE gene. In this study, a dual-luciferin reporting system was used to detect that pUL48 of DPV could enhance the activity of all IE gene ICP4, ICP22, and ICP27 promoters but did not affect the activity of E gene and L gene promoters (Figure 1). These results indicate that DPV pUL48, like the VP16 homologous protein of other herpesviruses, specifically acts on the α-TIF of IE gene and promotes the transcriptional activation of the IE gene. However, in addition to promoting gene transcription by activating the promoter of the IE gene, whether pUL48 can promote gene transcription by directly acting on the IE gene has not been studied in this paper, nor has it been reported in other α herpesviruses. Subsequently, whether pUL48 can directly interact with the IE gene can further influence its transcription level.

Other herpesviruses exhibit transcriptional activation by activating specific *cis*-elements upstream of the IE gene promoter, but the *cis*-elements are not fixed motifs. The upstream of all IE gene promoters of HSV-1 contain a specific sequence, TAATGARAT, to which VP16 binds via Oct-1 to activate the transcription of HSV-1 IE gene. Similar sequences to this specific motif may exist upstream of promoters of other α-herpesvirus IE genes, such as bovine herpesvirus type 1 (BHV-1); VP16 of BHV-1 can act on TAATGAGCT repeat motif upstream of ICP0 promoter or canine herpesvirus (CHV) TAATTACAC motif upstream of ICP4 promoter promotes transcriptional activation of BHV-1 ICP0 and CHV ICP4, respectively (Misra et al., 1994; Tyack et al., 2006). However, there is no similar motif recognized by VP16 in the upstream of other IE gene promoters of BHV-1 and CHV except ICP0 and ICP4. As a result, VP16 cannot activate their transcription, and the transcription of these IE genes is regulated by other transcription activators, such as the BHV-1 IE gene is mainly regulated by ICP0 (Kook et al., 2015), indicating that VP16, as the α-TIF of IE gene, cannot activate all IE gene transcriptions, which depend on whether there are *cis*-acting elements that fit VP16 upstream of its promoter. In this study, the dual-luciferase reporting system was first used to study the activation effect of pUL48 on ICP4-Pro (1,235 bp) was much higher than that on ICP4-Pro (740 bp) (Figure 2). These results suggest that there must be *cis*-elements in the upstream of ICP4 promoter between 740- and 1,235-bp sequences that highly fit the action of pUL48. Therefore, the upstream sequences of the ICP4 and ICP22 promoters of DPV were compared with *cis*-element sequences of VP16 reported by other herpesviruses, and possible *cis*-element sequences were found. The luciferin reporter plasmids with different length promoters of ICP4 and ICP22 were constructed according to the location of different *cis*-elements. The activity of ICP4-Pro (1,235 bp) increased nearly 2,000 times under pUL48 using double luciferin reporter system. The activity of ICP4-Pro (1,226 bp) without TAATGATAT at 1,227–1,235 bp was only increased by about 500 times. ICP4-pro (1,226 bp) and ICP4-Pro (900 bp) contain the TAATGTTAT sequence located at 748–756 bp and a different number of repeated sequences TAATTATAT, but there was no difference in the activity of these two sequences under pUL48, and ICP4-Pro (740 bp) activity was only increased several times, indicating that the TAATGATAT sequence at 1,227–1,235 bp upstream of the ICP4 promoter is the core *cis*-element wherein pUL48 promotes ICP4 transcriptional activation (Figure 3B).

Similarly, the sequence TAATGTTAT at 748–756 bp is also a *cis*-acting element of pUL48, but it is not the main one. The ICP22-Pro (400 bp) and ICP22-Pro (900 bp), containing TAATGTTAT at 303–311 bp upstream of the ICP22 promoter activity, were enhanced under pUL48; however, there was no difference between the two. ICP22-Pro (300 bp) could not be activated by pUL48 (Figure 3C), indicating that the sequence TAATTATAT was a *cis*-element of pUL48 acting on ICP22. Although there were five repeat units upstream of the ICP4 promoter in this sequence, it was not a *cis*-element of pUL48 acting on ICP4. These results indicated that even though there were the same DNA sequences recognized by α-TIF upstream of different gene promoters, not all of them could act, which might be affected by other *cis*-elements upstream of the promoters. The above three-element sequences did not exist upstream of the DPV ICP27 promoter, and the activation effect of pUL48 on the ICP27 promoter was significantly lower than that of ICP4 and ICP22, suggesting that other transcriptional activators mainly regulated the transcriptional activation of ICP27. This study did not confirm the binding of pUL48 to the DNA target sequence, and there may be other *cis*-element sequences that have not been screened. Gel block or chromatin immunoprecipitation experiments can further detect more *cis*-element sequence binding to pUL48 (Dugan et al., 2016).

Transcriptional activation of other herpesviruses VP16 is dominated by conserved DNA-binding domains (DBD) and non-conserved transcriptional activation domains (TAD). The TAD position, sequence, and amino acid properties of different VP16 homologies are very different. HSV, EHV, and CHV VP16 TAD are at the C-terminal, while BHV and Varicella-Zoster virus (VZV) VP16 TAD is at the N-terminal (Cohen and Seidel, 1994; Tyack et al., 2006). In this study, the homology of DPV pUL48 and other α herpesvirus VP16 homologies were compared, and the structure of DPV pUL48 is similar to that of VZV α-TIF, whose TAD was located at the N-terminal. Therefore, the N-terminal truncated protein of DPV pUL48 was constructed to explore the position of TAD. pUL48 without the N-terminal 1–60 aa lost the activation of the IE gene promoter (Figure 4), so DPV pUL48 TAD was located at N-terminal 1–60 aa.

A further study of the core domain of pUL48 TAD found that pUL48 with a 1− to 20-aa deletion at the N-terminal was significantly inhibited in activating the IE gene promoter, while pUL48 with a 21− to 40-aa deletion or 41− to 60-aa deletion only slightly decreased in activating the IE gene promoter (Figure 5), indicating that the N-terminal 1− to 20-aa was the core domain of pUL48 TAD, and the relative quantitative PCR results confirmed this conclusion (Figure 6). The key amino acid sites in the core TAD domain of pUL48 were not further explored in this paper. For example, the ETIF TAD of EHV-1 is the last seven amino acids, among which methionine (Met) is critical, and this amino acid is conserved in CHV and HSV-1 VP16 (Grapes and O’Hare, 2000). Met also exists in the TAD core domain of DPV pUL48, and whether it also has an important influence on the transcriptional activation function of DPV pUL48 needs further study.

In recent years, the binding of VP16 TAD to the target protein and the binding form of VP16 TAD has been studied. For example, when HSV-1 VP16 TAD binds to the transcription factor PC4, an α helix is formed in TAD, and TAD C terminal is responsible for providing an electrostatic driving force to enhance the binding of TAD N terminal to PC4. If induced mutation reduces the affinity of the TAD C terminal, the binding force of the TAD N terminal will be lost (Langlois et al., 2008). Some studies have also fused VP16 TAD into plant transcriptional suppressors, using this technology to explore the inhibitory function and mechanism of transcriptional suppressors to strongly activate “silent genes” that are not normally expressed (Aguilar et al., 2014; Fujiwara et al., 2014). Currently, more and more studies have shown that VP16 plays an important role in the reactivation of α herpesvirus from latency (Sawtell and Thompson, 2016, 2021). Ala replaced HSV-1 VP16 Ser375 to block the binding of VP16 to Oct-1, thus, inhibiting the transcriptional activation function of VP16. As a result, latently infected neurons reexpressing VP16 during heat shock were significantly reduced (Sawtell et al., 2011), indicating that VP16 transcriptional activation is critical for HSV-1 latent reactivation.

To explore the effect of other DPV viral proteins on the transcriptional activation function of pUL48, we found that pUL14, pUL46, and pUL47 could significantly enhance the activation of pUL48 on the promoter ICP4 and ICP22 of the IE gene through the detection of the dual-luciferin reporting system. However, neither one of them alone significantly affected ICP4 and ICP22 promoter activity (Figure 7). Since pUL14, pUL46, and pUL47 all have NLS, to determine whether the enhanced effect of these proteins on the transcriptional activation function of pUL48 is related to the influence on the localization of pUL48, this study used the eukaryotic expression plasmid of pUL47 and its two nuclear localization signals 40− to 50-aa/768− to 777-aa deletion plasmids to explore. The results showed that pUL48 was dispersed in the cytoplasm and nucleus and mainly located in the cytoplasm when transfected alone, while pUL47 could promote the nucleation of pUL48 through its NLS located at 40–50 aa/768–777 aa (Figure 8), which was further confirmed that after the loss of pUL47 NLS, activation of ICP4 promoter by pUL48 was not enhanced (Figure 8). Some literature (Zhang et al., 1991; Vittone et al., 2005; Ohta et al., 2011; Svobodova et al., 2012) reported that pUL14, pUL46, and pUL47 could participate in the process of VP16 entering the nucleus and regulate the *trans*-induction of VP16 after HSV-1-infected host cells. Some literature (Li et al., 2016) also reported that DPV pUL14 and pUL48 colocated in the nucleus.

Furthermore, pUL14 can promote the nucleation of pUL48. This study showed that pUL14, pUL46, and pUL47 of DPV could synergistically enhance the transcriptional activation function of pUL48 and promote the transcription of the IE gene by promoting the entry of pUL48 into the nucleus. The inhibitory effect of pUL49 on the transcriptional activation function of pUL48 has also been reported in HSV-1. HSV-1 pUL49 can act on transcriptional regulatory factors, such as VP16 and ICP0, to inhibit the activation of promoters (Yu et al., 2008). The mechanism is not clear, but HSV-1 pUL49 can directly bind to VP16 TAD. When pUL49 and VP16 are coexpressed without other viral proteins, they can colocate around the nuclear membrane (Wu et al., 2020). Therefore, we hypothesized that pUL49 of DPV might inhibit its transcriptional activation by preventing pUL48 from entering the nucleus, which could also be studied in the future.

## Data Availability Statement

The original contributions presented in the study are included in the article/supplementary material, further inquiries can be directed to the corresponding author.

## Author Contributions

TZ and DF carried out the experiments. AC and MW conceived of and supervised the study. MW, XO, SM, DS, SZ, QY, YW, RJ, DZ, SC, and ML contributed to the conception of this article. X-XZ, JH, QG, and BT helped to drew the images. MW modified the article. All authors reviewed the manuscript.

## Conflict of Interest

The authors declare that the research was conducted in the absence of any commercial or financial relationships that could be construed as a potential conflict of interest.

## Publisher’s Note

All claims expressed in this article are solely those of the authors and do not necessarily represent those of their affiliated organizations, or those of the publisher, the editors and the reviewers. Any product that may be evaluated in this article, or claim that may be made by its manufacturer, is not guaranteed or endorsed by the publisher.
